# Concurrent guiding of light and heat by transformation optics and transformation thermodynamics via soft matter

**DOI:** 10.1038/s41598-018-29866-w

**Published:** 2018-07-30

**Authors:** Wallysson K. P. Barros, Erms Pereira

**Affiliations:** 1 Polytechnic School of Pernambuco, Universidade de Pernambuco, Rua Benfica, 455, 50720-001 Recife, PE Brazil; 2 Departament of Physics, Universidade Federal Rural de Pernambuco, 52171-900 Recife, PE Brazil

## Abstract

Controlling light and heat via metamaterials has presented interesting technological applications using transformation optics (TO) and transformation thermodynamics (TT). However, such devices are commonly mono-physics and mono-purpose, because the used metamaterial is designed to deal with one type of physical mechanisms. Here we demonstrate, for the first time, how to connect TO and TT via the liquid crystal 4-Cyano-4’-pentylbiphenyl (5CB) and, to exemplify such link, we present a multiphysics, multi-purpose device that simultaneously controls light and heat using such material. The anisotropic multiphysics properties of 5CB bond TO and TT, expanding the usage of these theories. The device, composed by 5CB confined between two right circular concentric cylinders, concentrates light (as a converging lens) and simultaneously repels heat from the inner cylinder when the molecules are along the direction $\hat{{\boldsymbol{\rho }}}$ and it disperses light (as a diverging lens) and concurrently concentrates heat to the inner cylinder, without disturbing the external temperature field, when the molecules are along the direction $\hat{{\boldsymbol{\theta }}}$, contributing for saving materials and designing miniaturized multiphysics systems.

## Introduction

Transformation optics (TO) and transformation thermodynamics (TT) has presented many technological possibilities in the last years. We have many examples, as black hole analogues^1,2^, cloaking devices^3–5^, perfect lenses^6,7^, etc. These are done by devising effective distortions of the plane space (represented by coordinates transformations and an effective metric tensor) to achieve the properties of interest. The effective distortions are realized using metamaterials^8–10^. They are engineered materials formed by periodic structures of ordinary materials, where the dimensions of such structures are below the wavelength of the wave they manipulate.

Recently metamaterials are used to construct biosensor platform^11^, acoustic circulators^12^ and enhancers of pressure sound waves^13^. At this point, some problems rise. One is the metamaterial used deals with one kind of propagation of energy: or diffusion^14–16^ (thermal and electric) or wave^17^ (electromagnetic radiation and sound) equations. Another practical problem is that such metamaterials are in solid state, preventing changes on the material properties by an external controlling.

Following such ideas, this work presents, as its main result, the soft matter nematic liquid crystal^18,19^ as a system that naturally connects the transformation optics (TO) and the transformation thermodynamics (TT). We review the coordinate transformation properties of TO and TT, highlighting how the effective metric tensor produces the aimed permittivity or thermal conductivity tensor. After that, we present the main properties of nematic liquid crystals and its algebraic expression to obtain the permittivity and the thermal conductivity tensors from a spatial configuration of the liquid-crystalline molecules. Then we demonstrate how an unequivocal spatial molecular field of the liquid crystals is related to the same effective metric tensor obtained by TO and TT, linking both theories. To exemplify such union, we present a device that simultaneously controls the propagation of light and heat in an omnidirectionally way, realizing simulations from a finite element software.

## Model and Formalism

### Transformation optics and transformation thermodynamics

For achieving the desired permittivity and thermal conductivity tensors, TO^20,21^ and TT^3,22^ are based on continuous coordinate transformations from an isotropic material with a flat metric tensor of components *δ*_*ij*_, to a deformed one with *g*_*ij*_ the components of the new metric.

In Cartesian coordinates, the permittivity of the transformed media has the components^23^ ${\varepsilon }_{^{\prime} ij}={|{\rm{\det }}({{\rm{\Lambda }}}_{i^{\prime} }^{i})|}^{-1}$ ${{\rm{\Lambda }}}_{i^{\prime} }^{i}{{\rm{\Lambda }}}_{j^{\prime} }^{j}{\varepsilon }_{0}{\delta }_{ij}$, while the thermal conductivity tensor of the transformed media is given by ${\lambda }_{^{\prime} ij}={|{\rm{\det }}({{\rm{\Lambda }}}_{i^{\prime} }^{i})|}^{-1}{{\rm{\Lambda }}}_{i^{\prime} }^{i}{{\rm{\Lambda }}}_{j^{\prime} }^{j}{\lambda }_{0}{\delta }_{ij}$, where *ε*_0_ and *λ*_0_ are the permittivity and the thermal conductivity of the crystal phase, ${{\rm{\Lambda }}}_{i^{\prime} }^{i}=\partial {x}^{i}/\partial {x}^{i^{\prime} }$ are the components of the Jacobian matrix of the coordinate change, related to the curved metric tensor by ${g}_{^{\prime} ij}={|{\rm{\det }}({{\rm{\Lambda }}}_{i^{\prime} }^{i})|}^{-1}{{\rm{\Lambda }}}_{i^{\prime} }^{i}{{\rm{\Lambda }}}_{j^{\prime} }^{j}{\delta }_{ij}$. This implies that, if one has the metric tensor ${g}_{^{\prime} ij}$, the permittivity and thermal conductivity tensors are found by the relation^23–25^

1 $$\{\begin{array}{c}{\varepsilon }_{^{\prime} ij}\\ {\lambda }_{^{\prime} ij}\end{array}\}=\{\begin{array}{c}{\varepsilon }_{0}\\ {\lambda }_{0}\end{array}\}{g}_{^{\prime} ij}.$$

### Liquid crystals

Liquid crystals are materials composed by anisotropic molecules that has stable phases between the isotropic liquid and the solid crystal^18,19^ and they have good application for display^26^ and non-display usages^2,27–29^ of these phases is the nematic one, where the molecules (rod-like, in this work) are randomly positioned in the space, but they have an average molecular orientation along a local molecular field $\hat{n}$ called *director*. One possibility on disturbing such molecular field is the presence of topological defects of the nematic phase, that, in fact, they can be present in different physical systems^30–32^. Depending on the direction of $\hat{n}$, one can enhance the anisotropy of different macroscopic properties: thermal^24,27,28^, optics^2,33–35^, acoustics^19,36^, etc.

Many anisotropic properties of the nematic phase of liquid crystals are related to the local molecular director $\hat{n}$^18,36,37^. For a given $\hat{n}$, the components *λ*^*ij*^ of the thermal conductivity tensor are given, in Cartesian coordinates, by^37^

2 $${\lambda }_{ij}={\lambda }_{iso}{\delta }_{ij}+{\lambda }_{a}({n}_{i}{n}_{j}-\frac{{\delta }_{ij}}{3}),$$

being ${\lambda }_{iso}=\frac{{\lambda }_{\parallel }+2{\lambda }_{\perp }}{3}$, ${\lambda }_{a}={\lambda }_{\parallel }-{\lambda }_{\perp }$ and the parallel and perpendicular molecular thermal conductivities are ${\lambda }_{\parallel }$ and *λ*_⊥_. It is reasonable to regard the temperature dependence of the molecular thermal conductivities. Thus, for the liquid crystal 5CB, we have^38^

3 $${\lambda }_{\parallel }(T)={\lambda }_{0}+{\lambda }_{1}\cdot (T-{T}_{NI})+{\lambda }_{1,\parallel }{({T}_{C}-T)}^{{\alpha }_{\parallel }},$$

4 $${\lambda }_{\perp }(T)={\lambda }_{0}+{\lambda }_{1}\cdot (T-{T}_{NI})+{\lambda }_{\mathrm{1,}\perp }{({T}_{C}-T)}^{{\alpha }_{\perp }},$$

where *λ*_0_, *λ*_1_, ${\lambda }_{\mathrm{1,}\parallel }$, ${\alpha }_{\parallel }$ and *α*_⊥_ are material constants and *T*_*NI*_ and *T*_*C*_ are, respectively, the nematic-to-isotropic temperature and the clearing-point temperature of the regarded liquid crystal.

On the electromagnetic phenomena and disregarding absorption, whether such absorption is weak for 5CB^39^ or it doesn’t rule the deflection of light, the components of the permittivity tensor for the nematic phase of liquid crystals are given by^37^

5 $${\varepsilon }_{ij}={\varepsilon }_{iso}{\delta }_{ij}+{\varepsilon }_{a}({n}_{i}{n}_{j}-\frac{{\delta }_{ij}}{3}),$$

being ${\varepsilon }_{iso}=\frac{{\varepsilon }_{\parallel }+2{\varepsilon }_{\perp }}{3}$, ${\varepsilon }_{a}={\varepsilon }_{\parallel }-{\varepsilon }_{\perp }$ and the parallel and perpendicular molecular permittivity constants are ${\varepsilon }_{\parallel }$ and *ε*_⊥_. For simplicity and based on the comparison between our obtained results shown in the next section and the published results using ray optics^5^, we regard ${\mu }_{ij}={\mu }_{iso}{\delta }_{ij}+{\mu }_{a}({n}_{i}{n}_{j}+{\delta }_{ij}\mathrm{/3})\approx Diag({\mu }_{0})$ for the components of the permeability tensor. For the liquid crystal 5CB, the temperature dependences of the permittivity tensor can be related to the ones of the molecular refractive indexes by^40^

6 $${n}_{\parallel }(T)\approx A-BT+\frac{2{({\rm{\Delta }}n)}_{0}}{3}{(1-\frac{T}{{T}_{c}})}^{\gamma },$$

7 $${n}_{\perp }(T)\approx A-BT-\,\frac{{({\rm{\Delta }}n)}_{0}}{3}{(1-\frac{T}{{T}_{c}})}^{\gamma },$$

where *A*, *B* and *γ* are material constants, (Δ*n*)_0_ is the birefringence when in crystal phase and *T*_*C*_ is the clearing-point temperature of the regarded liquid crystal.

Due to the birefringence, the refractive index of the extraordinary ray of the group velocity of an electromagnetic wave inside the nematic phase is given by^41^

8 $${N}_{g}^{2}={n}_{\parallel }^{2}{\sin }^{2}\beta +{n}_{\perp }^{2}{\cos }^{2}\beta ,$$

where $\beta =\arccos \,(\hat{n}\cdot \overrightarrow{S})$, with $\overrightarrow{S}$ being the Poyting vector.

## Results and Discussion

### **C**onnection between TO and TT

Differently of solid metamaterials, that are designed to work for a specified type of energy, nematic liquid crystals are anisotropic materials for simultaneous types of energy. This happens because the similarity between the thermal conductivity tensor, Eq. (2), and the permittivity tensor, Eq. (5), producing the same effective metric tensor by Eq. (1), resulting in

9 $$\{\begin{array}{c}{\varepsilon }_{^{\prime} ij}\\ {\lambda }_{^{\prime} ij}\end{array}\}=\{\begin{array}{c}{\varepsilon }_{iso}{\delta }_{ij}+{\varepsilon }_{a}({n}_{i}{n}_{j}-\frac{{\delta }_{ij}}{3})\\ {\lambda }_{iso}{\delta }_{ij}+{\lambda }_{a}({n}_{i}{n}_{j}-\frac{{\delta }_{ij}}{3})\end{array}\}=\{\begin{array}{c}{\varepsilon }_{0}\\ {\lambda }_{0}\end{array}\}{g^{\prime} }_{ij}.$$

Such metric tensor, that TO and TT require do modify the material properties, can be achieved by a unique molecular director configuration $\hat{n}(\overrightarrow{r})$ of nematic liquid crystals, connecting both theories. For example, the molecular directors $\hat{n}=\hat{\rho }$ and $\hat{n}=\hat{\theta }$ in cylindrical coordinates arise the following metric tensor^24^:

10 $${(g)}_{ij}=(\begin{array}{cc}1 & 0\\ 0 & {\beta }^{2}{\rho }^{2}\end{array}),$$

where ${\beta }^{2}=\frac{{\lambda }_{\perp }}{{\lambda }_{\parallel }} < 1$ for $\hat{n}=\hat{\theta }$ and ${\beta }^{2}=\frac{{\lambda }_{\parallel }}{{\lambda }_{\perp }} > 1$ for $\hat{n}=\hat{\rho }$. We substitute the metric (10) in (9) to obtain the components of the material tensors or the components of the molecular field $\hat{n}$, where the thermal conductivity tensor is explicitly

11 $$\begin{array}{rcl}{(\lambda )}_{ij} & = & {\lambda }_{0}{(g)}_{ij},\\  & = & {\lambda }_{0}(\begin{array}{cc}1 & 0\\ 0 & {\beta }^{2}{\rho }^{2}\end{array}),\\  & = & (\begin{array}{cc}{\lambda }_{\rho \rho } & 0\\ 0 & {\lambda }_{\theta \theta }\end{array}).\end{array}$$

The thermal conductivities *λ*_*ρρ*_ and *λ*_*θθ*_ are, respectively, the ones of the radial and polar directions. For $\hat{n}=\hat{\theta }$, we have ${\lambda }_{\rho \rho }={\lambda }_{\parallel }$ and *λ*_*θθ*_ = *λ*_⊥_; while for $\hat{n}=\rho$, we have *λ*_*ρρ*_ = *λ*_⊥_ and ${\lambda }_{\theta \theta }={\lambda }_{\parallel }$.

Observe that, from now on, any nematic liquid crystal described by Eq. (9) can compose devices that are naturally multiphysics ones. To give only one example of connection between TT and TO, we present in the next subsection a device that displays a simultaneous omnidirectional controlling of the propagation of heat and electromagnetic waves using the nematic liquid crystal 5CB. To highlight such connection, we regard the temperature dependence of the molecular thermal conductivities using the Eqs (3) and (4) and the refractive indexes by Eqs (6) and (7).

### Simulations

**Figure 1:**
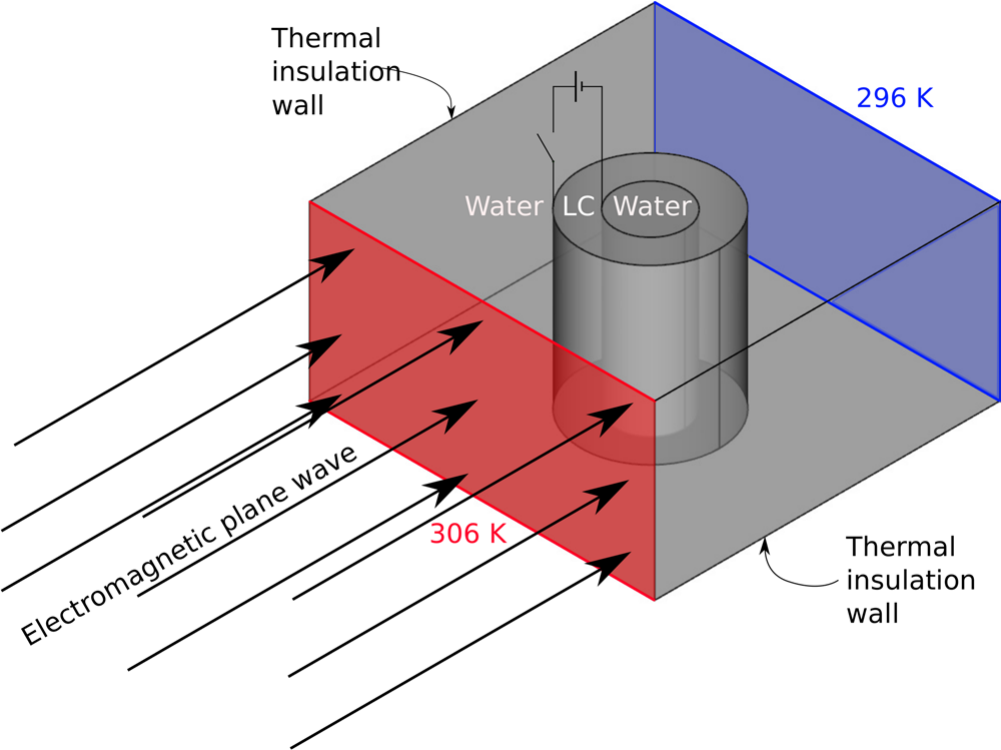
Scheme of the bi-objective multiphysics switchable controller in this paper. The liquid crystal (LC) 5CB is kept between two concentric cylinders, with water in the other regions. Adapted by permission from Springer Customer Service Centre GmbH: Nature Springer, The European Physical Journal E - Soft Matter, Sébastien Fumeron, Fernando Moraes, Erms Pereira, 2018.

For the proposed device, the Laplace and Fourier equations for the propagation of heat and the Maxwell equations for the propagation of electromagnetic waves will be solved using a finite element software (COMSOL Multiphysics). The artifact is composed of concentric cylinders, with the 5CB liquid crystal^38,40,42^ filling the region between them and the same material (here it was water, where we are disregarding absorption) filling the regions in the inner cylinder and out the outer one, as in Fig. 1. The structure of concentric cylinders keeping liquid crystal has been experimentally known for a long time^43,44^ and studied exhaustively^45–47^. This device was analyzed with the molecular director $\hat{n}$ in two different directions: in the radial direction $\hat{n}=\hat{\rho }$ and in the azimuthal direction $\hat{n}=\hat{\theta }$, with such directors switched by, for example, an external electric field via the Freedericks transition^18,37^. One can prepare these directors, for instance^48^, coating the exterior surface of the inner cylinder with indium tin oxide (ITO), setting a homeotropic strong anchoring energy, and the interior surface of the outer cylinder with Polymethylmethacrylate, producing a weak anchoring energy. Because the device has symmetry for each plane orthogonal to the axis of the concentric cylinders, we model cross-section views of these cylinders in a right rectangular prism. Such model consists on a rectangle with dimensions 200 × 120 *μm* containing on its center two concentric circles with radii 42.25 *μm* and 20 *μm*. These geometric dimensions can avoid convection in the liquid crystal for the applied temperature gradient, as can be seen in details in^37^. The region between the circles is filled with the 5CB liquid crystal, while water fills the region in the inner cylinder and between the outer cylinder and the rectangle. For the rectangle, we set fixed temperatures at 296 *K* and 306 *K* on opposite 120 *μm* sides, while we define insulating walls for the other ones. We use a finite element software to simulate the propagation of heat and radio-frequency (RF) electromagnetic wave in such device, with both molecular thermal – Eqs (3) and (4) – and dielectric – Eqs (6) and (7) – properties depending on the temperature.

#### The thermal case

**Figure 2:**
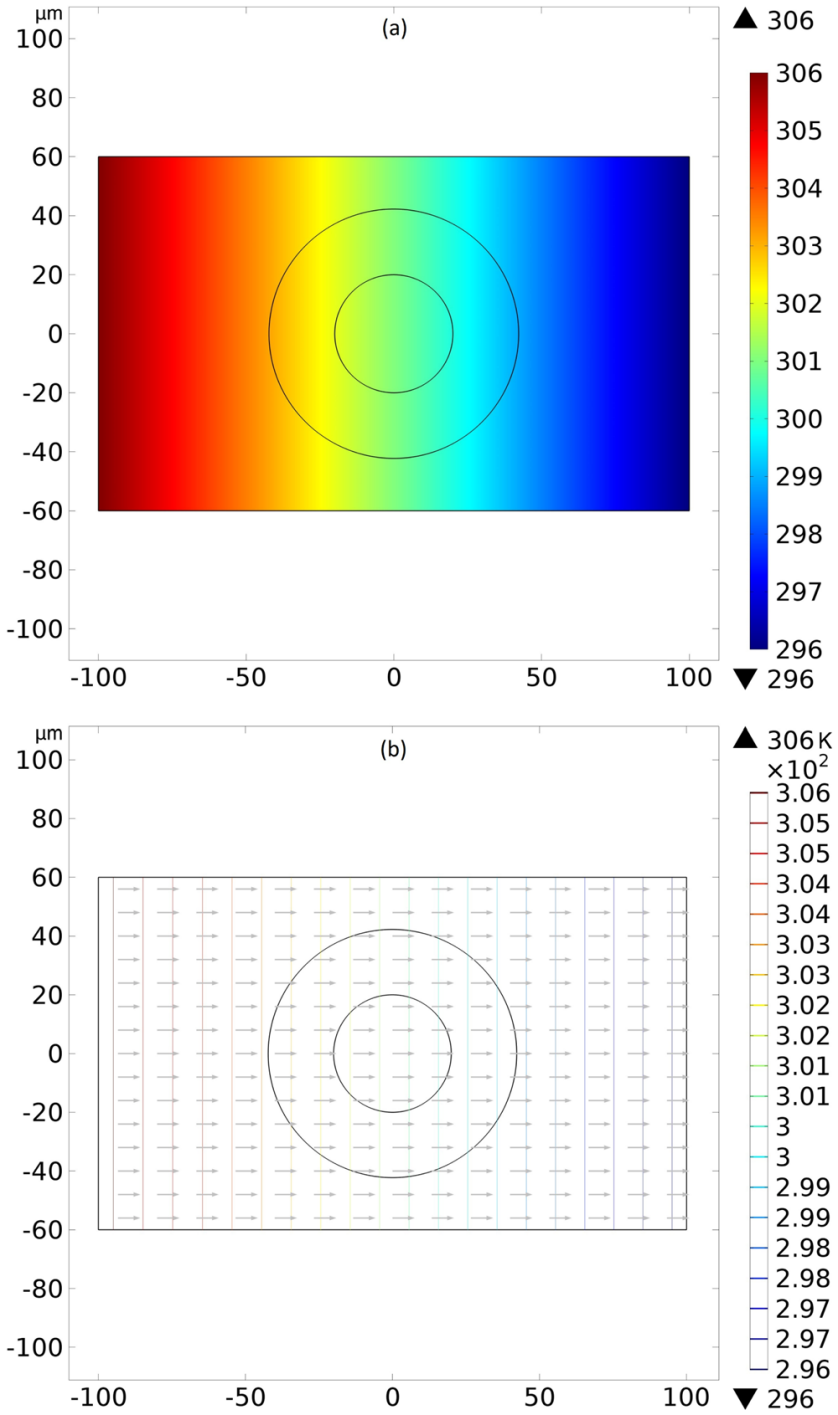
Axial transversal view of the temperature field (**a**) and the isothermal surfaces (**b**) created by the water everywhere for reference purposes. Colors represent temperatures in kelvins.

**Figure 3:**
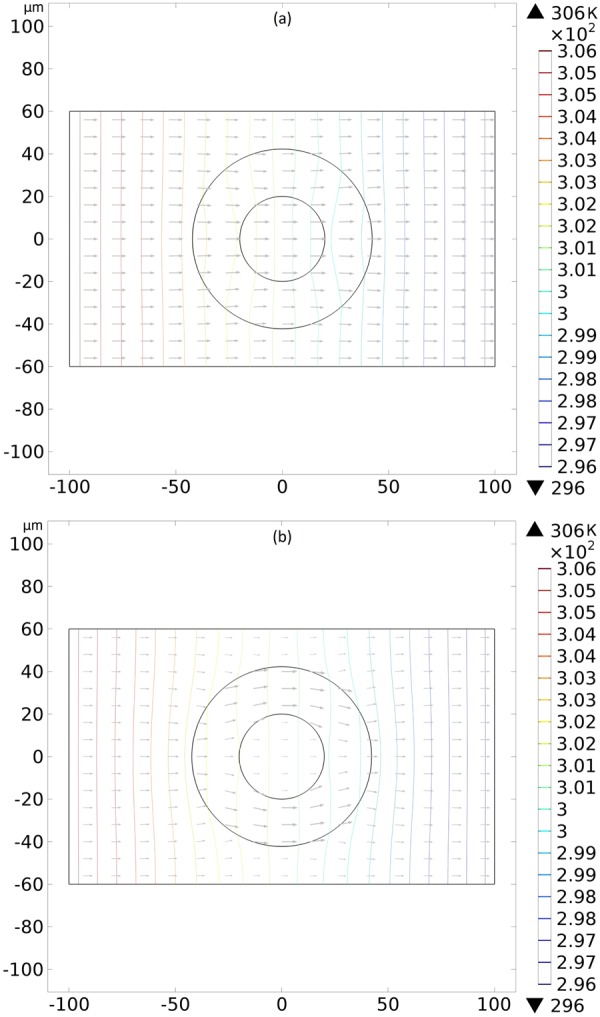
Axial transversal view of the isothermal surfaces and the heat flux vectors created by the liquid-crystalline controller. In (**a**), the concentration of isothermal lines is observed inside the inner cylinder, not disturbing the external thermal field. Here, the molecular director $\hat{n}$ between the cylinders is in the azimuthal direction $\hat{\theta }$. In (**b**) we have the repulsion of the isothermal lines from the inner cylinder, disturbing the external thermal field, with the molecular director $\hat{n}=\hat{\rho }$ between the cylinders. Colors represent temperatures in kelvins.

A better understand of the action of the device on the thermal phenomena is acquired determining the isothermal surfaces (solving the Laplace equation) and the heat flux vectors (solving the Fourier equation) for the two work modes of our controller. We present a reference case with water everywhere in Fig. 2 and the effects of our device in Fig. 3. In Fig. 3(a), we have the molecular director $\hat{n}=\hat{\theta }$ and the isothermal surfaces between the cylinders are deformed to increase the temperature gradient inside the inner cylinder. Another important aspect on this Fig. 3(a) is that the temperature field outside of the device is not deformed, meaning that an outside observer measuring only its local temperature field is unable to detect the presence of the device, characterizing a cloaked thermal concentrator. In Fig. 3(b), we have the director $\hat{n}=\hat{\rho }$ and the isothermal surfaces between the cylinders and outside of the controller are deformed to decrease the temperature gradient inside the inner cylinder, representing a thermal repeller. One can comprehend the thermal concentration and repelling of the controller from the ideas of transformation thermodynamics^3,49^. Among the needed conditions that the conductivity tensor must satisfy to produce a cloaked (i.e., without disturbing the exterior temperature field) concentrator and a cloaked repeller, we have, respectively, the following ones:

12 $${\lambda }_{\rho \rho } > {\lambda }_{\theta \theta },$$

13 $${\lambda }_{\rho \rho } < {\lambda }_{\theta \theta }.$$

**Figure 4:**
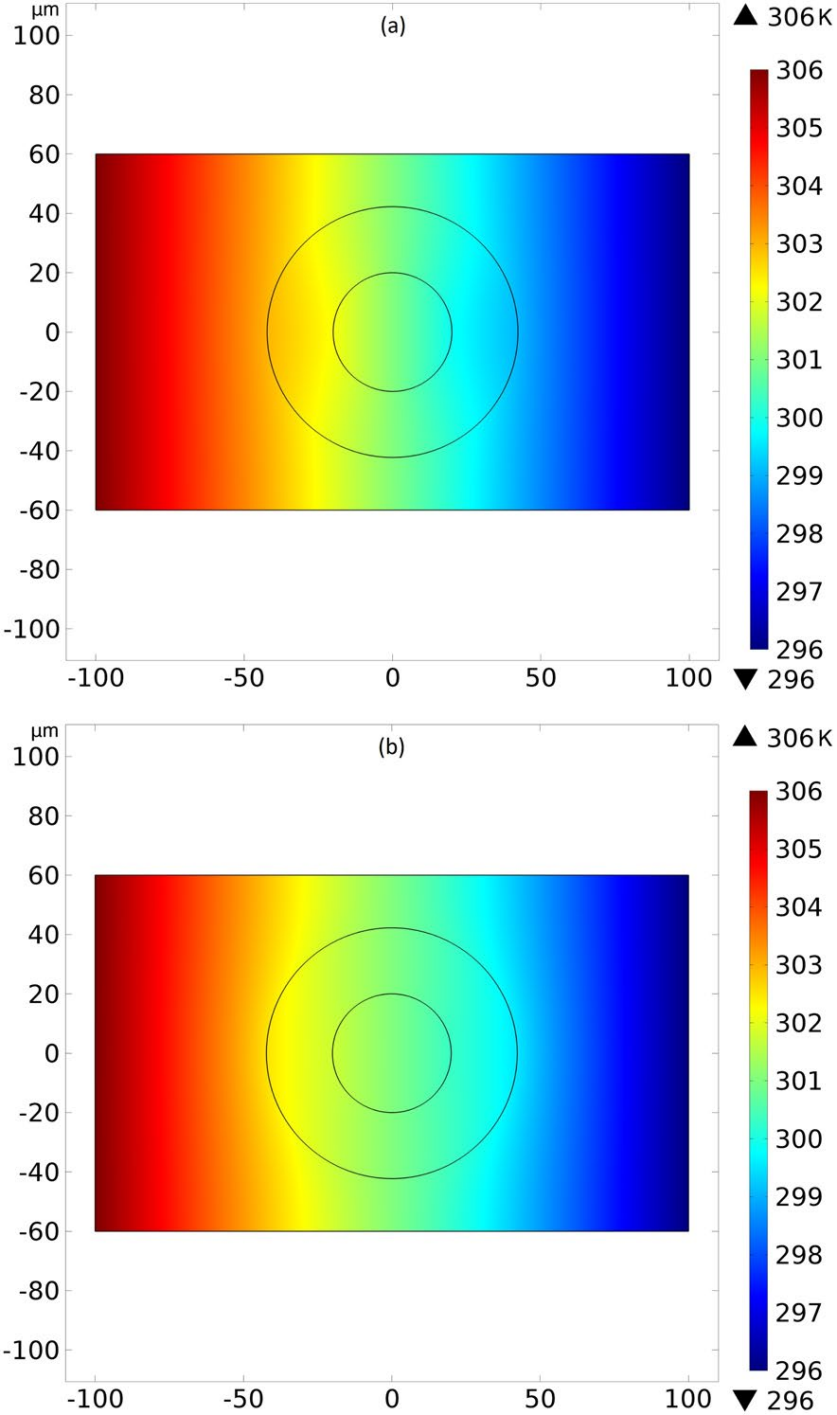
Axial transversal view of the temperature field created by the liquid-crystalline controller. In (**a**), the molecular director $\hat{n}$ between the cylinders is in the azimuthal direction $\hat{\theta }$. One notices a high gradient of temperature is observed inside the inner cylinder, not disturbing the external thermal field. In (**b**), with the molecular director $\hat{n}=\hat{\rho }$ between the cylinders, we have a low gradient of temperature inside the inner cylinder, disturbing the external thermal field. Colors represent temperatures in kelvins.

The Eq. (11) shows that the condition (12) for the concentrator is met in our device when $\hat{n}=\hat{\theta }$, while the condition (13) for the repeller is not met in our device when $\hat{n}=\hat{\rho }$. In the works^3,27,50^, the thermal conductivities were considered constants. In our simulations, we have used the temperature nonlinear equations for the molecular thermal conductivities (12) and (4) and we found similar qualitative results. Thus, Eqs (12) and (13) are approximate conditions for the cloaked effects, justifying the distortions in Figs 3 and 4, with the real ones can be found in^3^. This means that the controller concentrates or repels the heat flux even when the thermal conductivities depend on the temperature.

The action of the controller on the temperature field was calculated and it is shown in Fig. 4. We observe the concentration of heat through the inner cylinder in Fig. 4(a) without disturbing the external temperature field, and in Fig. 4(b) we observe the repelling of heat from the inner cylinder, perturbing the external temperature. Again, we have results similar to the ones in^3,27,50^. Having the value of the temperature for each spatial position, we link the action of this device on the thermal propagation to the propagation of RF wave by temperature dependent refractive indexes, Eqs (6) and (7).

#### The electromagnetic case

**Figure 5:**
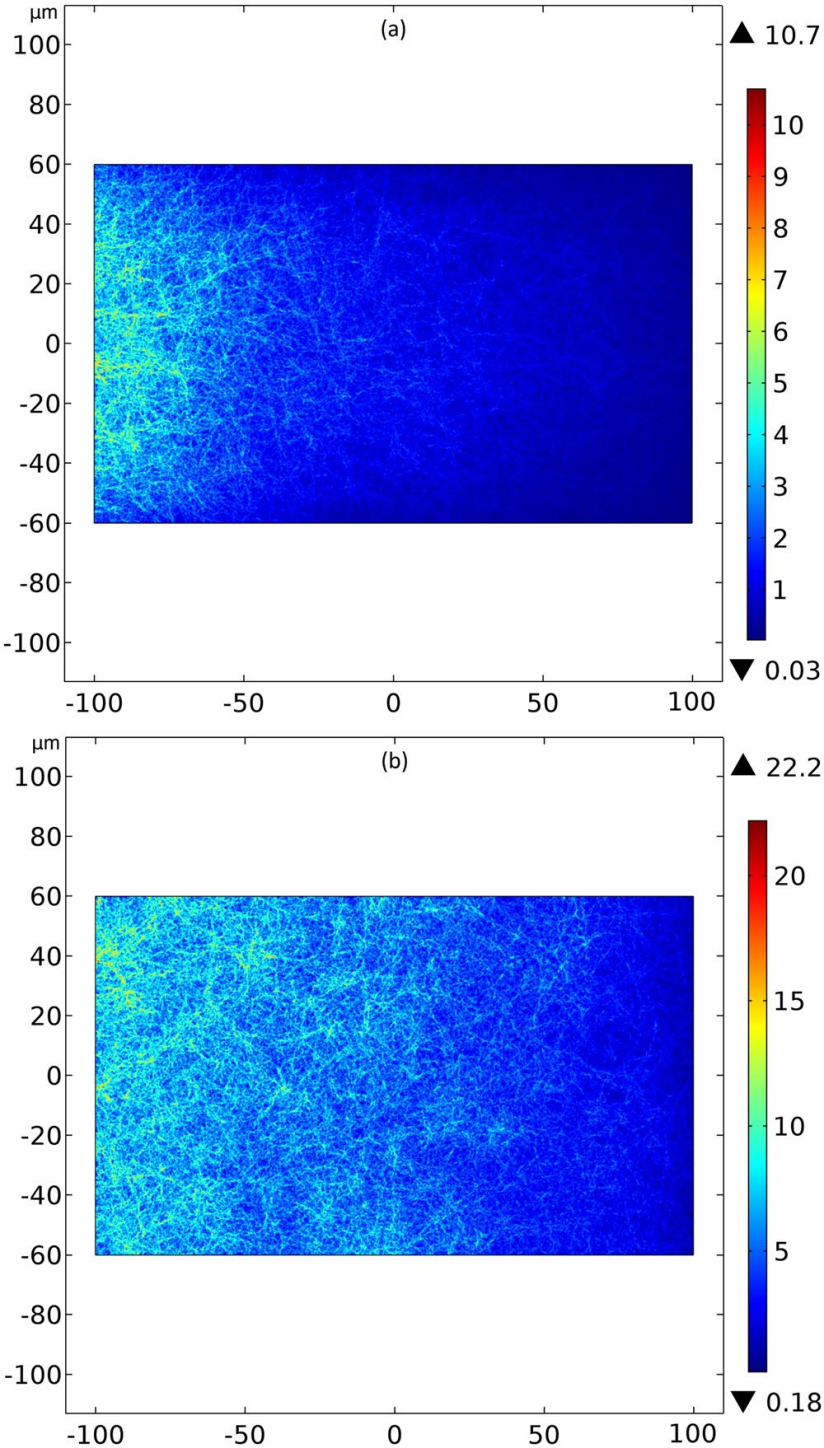
Intensity of the electric field for an incoming left-to-right RF wave with wavelength of 4.48 *μm*, electric field amplitude of 1 *V*/*m* and water everywhere, for reference purposes. The upper and lower horizontal walls are perfect absorbers and perfect conductors, respectively, in the pictures (**a**) and (**b**). Colors represent the intensity of the electric field in volts per meter.

**Figure 6:**
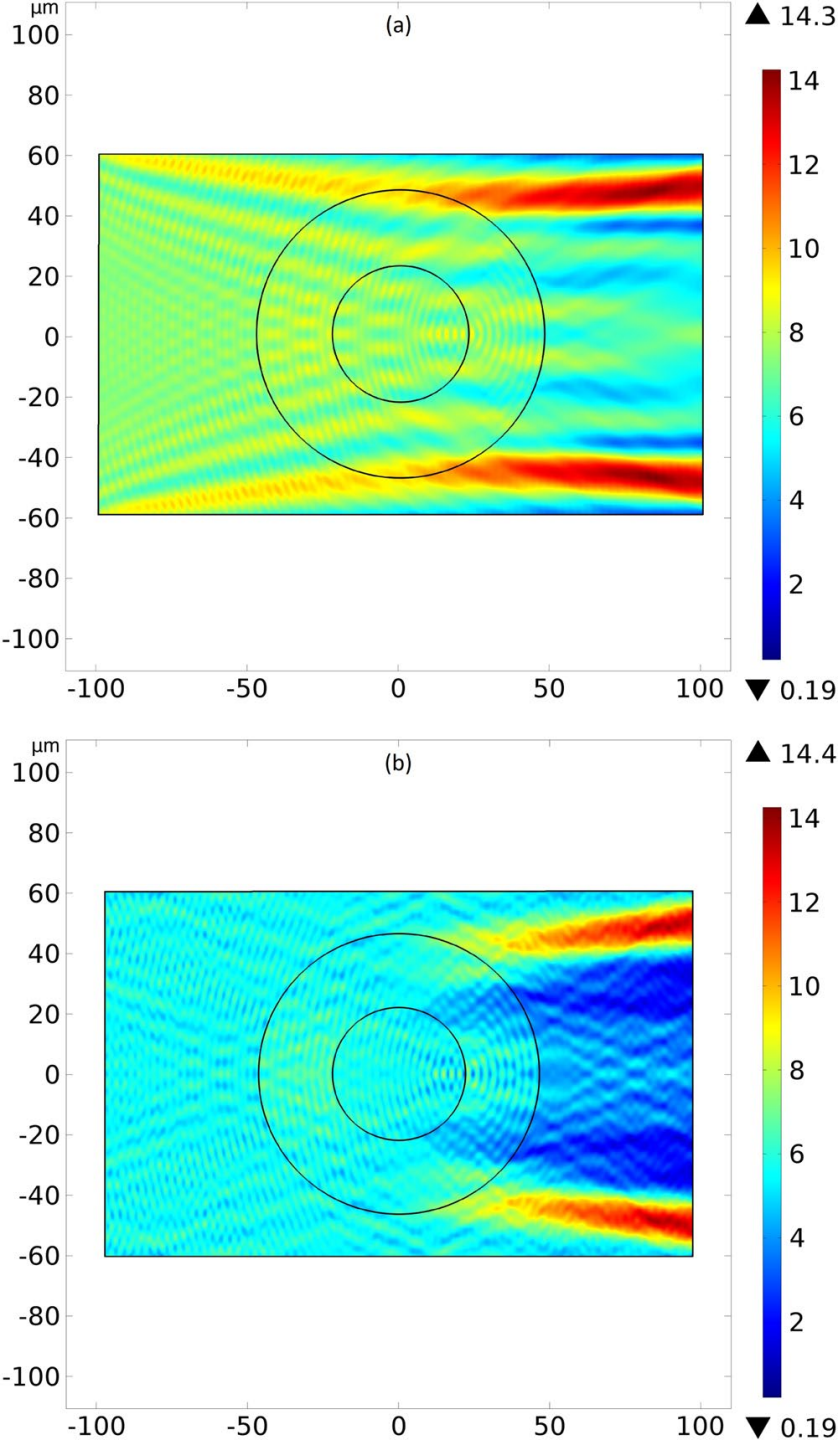
Intensity of the electric field for an incoming left-to-right RF wave with wavelength of 4.48 *μm*, electric field amplitude of 1 *V*/*m* and molecular director between the cylinders $\hat{n}=\hat{\rho }$. The upper and lower walls are perfect absorbers and perfect conductors, respectively, in the pictures (**a**) and (**b**). Colors represent the intensity of the electric field in volts per meter.

**Figure 7:**
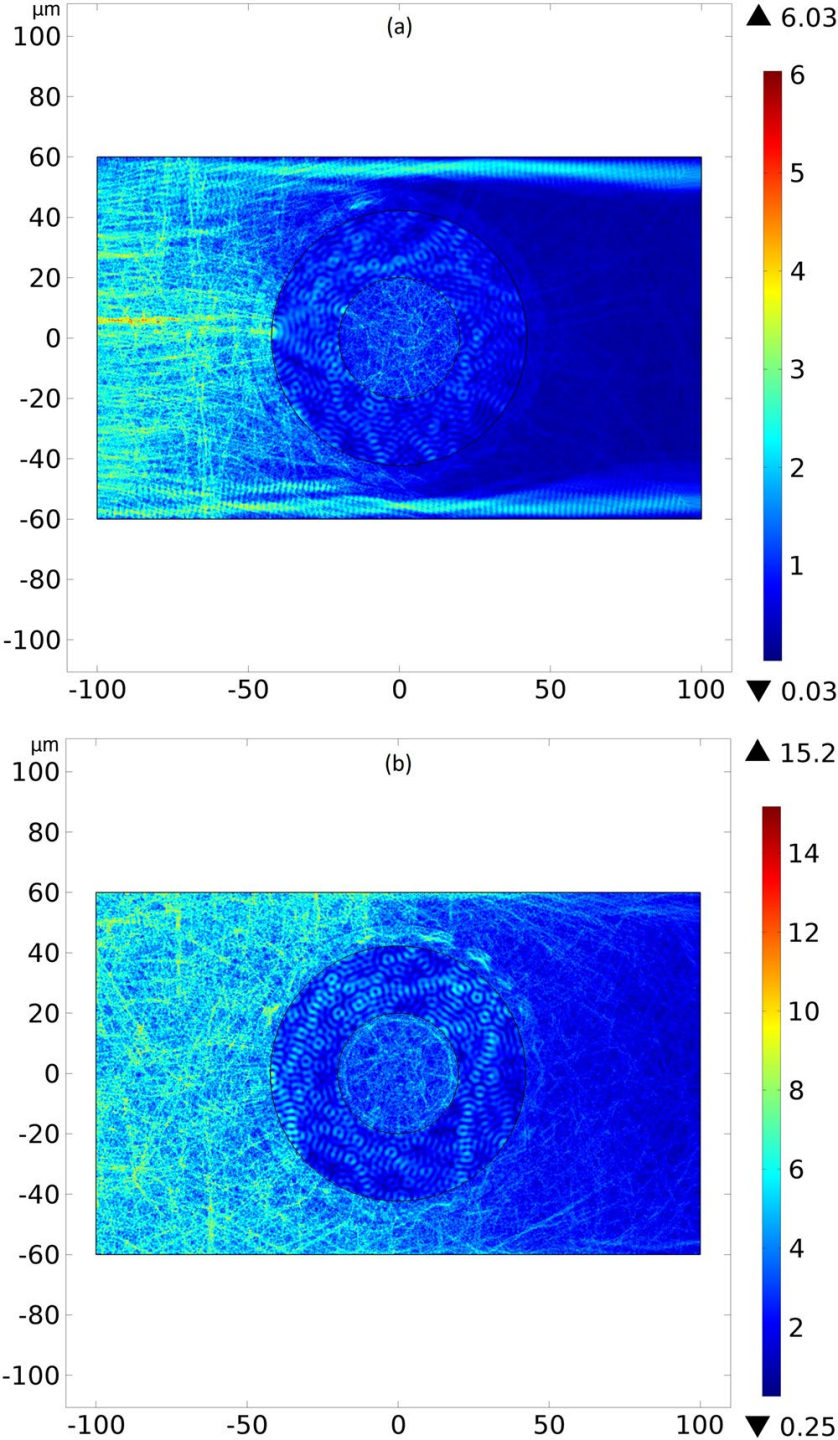
Intensity of the electric field for an incoming left-to-right RF wave with wavelength of 4.48 *μm*, electric field amplitude of 1 *V*/*m* and molecular director between the cylinders $\hat{n}=\hat{\theta }$. The upper and lower walls are perfect absorbers and perfect conductors, respectively, in the pictures (**a**) and (**b**). Colors represent the intensity of the electric field in volts per meter.

How the controller regulates the propagation of the RF waves due to the temperature dependence of the material properties is a critical matter on the designing of the device. And since the molecular director $\hat{n}$ determines the refractive index *N*_*g*_ by Eq. (8) and, consequently, the dielectric tensor $\overleftrightarrow{\varepsilon }$, one can also change at will the propagation of the RF waves by changing the orientation of $\hat{n}$ applying an electric and/or magnetic field. Solving the electromagnetic wave equation for $\hat{n}=\hat{\rho }$ and $\hat{n}=\hat{\theta }$, we found the intensity of the electric field. For Figs 5, 6 and 7, we have electromagnetic plane waves with wavelength of 4.48 *μm* and electric field amplitude of 1 *V*/*m* moving from the left vertical face to the right vertical face (a perfect absorber boundary) of the device. For figures (a), the upper and lower walls are perfect absorbers, while, for figures (b), such walls are perfect conductors.

As a reference case of the simulation, we have Fig. 5 with water everywhere. Since the planar wave comes from all the left boundary, we observe a scattering of the wave and an attenuation of the electric field intensity along the propagation of the wave, where we also have the electromagnetic absorption from the water.

In Fig. 6, the director is $\hat{n}=\hat{\rho }$ and we noticed a concentration of RF waves after its passage by the device. For Fig. 6(a), the perfect absorbers horizontal wall creates two symmetrical “paths” of high intensity light, while in Fig. 6(b) such two regions appear after the passage of the light through the device. The creation of these regions of high intensity of light has two equivalent interpretation: by Fermat principle^26,51^ or by the effective curved space where the liquid crystal lies^2,5,33^. On the latter geometrical approach, the light path represents a geodesic of such effective space, with a distorted trajectory in the region with the liquid crystal. Thus, the light in the liquid crystal describes paths between two spatial points A and B that minimizes the integral

$${\int }_{A}^{B}{N}_{g}dl={\int }_{A}^{B}dl\sqrt{\sum _{i,j=(x,y,z)}{g}_{ij}\frac{d{x}^{i}}{dl}\frac{d{x}^{j}}{dl}},$$

where *l* is a parameter along the path and *g*_*ij*_ are the components of the metric tensor^23^. Although this description is more suitable for ray optics^52^, our wave optics simulations, with the simplification *μ*_*ij*_ ≈ *Diag*(*μ*_0_), produced results similar to those in^5^, where the ray optics was used–being the approximation *μ*_*ij*_ ≈ *Diag*(*μ*_0_) reasonable^41^–and the temperature dependence for the molecular refractive indexes were not considered. Therefore, this indicates the device sustains its purpose even under such thermal link. The absence of the two regions of high intensity light before the device for the perfect conductor horizontal wall in Fig. 6(b) is an indication of destructive interference phenomena due to the reflection of the waves. Thus, when the molecular director is $\hat{n}=\hat{\rho }$, the device behaves like a converging lens, concentrating light that emerges from it.

In Fig. 7, the director is $\hat{n}=\hat{\theta }$ and we noted a relevant reducing of the emerging RF waves from the device for both kinds of horizontal walls, when comparing to Fig. 5. Again this event comes from the deflection of light by the liquid crystal between the cylinders with molecular director $\hat{n}=\hat{\theta }$. Such deflections can be interpreted, once more, by the effective curvature felt by light^2,52^. The decreasing on the intensity of the wave is according to results published in^5^. As a last comment, the annular regions around the boundaries of the concentric cylinders are consequence of the spatial meshing of the finite element simulation. Thus, when the molecular director is $\hat{n}=\hat{\theta }$, the device behaves like a diverging lens, dispersing light that emerges from it.

Observe that, when $\hat{n}=\hat{\rho }$, heat is repelled from the inner cylinder and the RF waves is concentrated after emerging from the device. The opposite situation takes place when $\hat{n}=\hat{\theta }$, where heat is concentrated on the inner cylinder, without perturbing the exterior temperature field, and RF waves is dispersed after emerging from the device. Such opposite pictures come from the different physics equations that were solved: diffusion equation for heat and wave equation for light.

## Summary and Conclusions

In this article, we studied how coexisting heat flux and electromagnetic waves are controlled by liquid crystal in its nematic phase, confined between concentric cylinders. Such thermal coupling with the propagation of the electromagnetic wave was made through the temperature dependence of the liquid crystal refractive indexes ${n}_{\parallel }$ and *n*_⊥_, using Eqs (6) and (7)^40^. For the molecular field $\hat{n}=\hat{\rho }$, the controller repels heat from the inner cylinder and it focuses the electromagnetic radiation to two strips of very high intensity emerging from the device. For the molecular field $\hat{n}=\hat{\rho }$, the controller concentrates heat to the inner cylinder without perturbing the exterior temperature field and decreases the intensity of the emerging electromagnetic radiation from the device. This is due to the fact that we are dealing with two energy transport regimes: thermal diffusion and wave propagation. An approach to understand such results is that the molecular fields $\hat{n}=\hat{\rho }$ and $\hat{n}=\hat{\theta }$ creates an effective curved space between the concentric cylinders, disturbing the propagation of heat and light in such region^5,24,27,33^. Although we considered the temperature dependence on the refractive indexes^40^ and on the molecular thermal conductivities^38^, our reported results are similar to the ones without such considerations^5,27^. This indicates a kind of thermal robustness of the proposed multiphysics controller and it can be a manifestation of the topological invariance of the physical properties of the liquid crystal phase. We say “topological invariance” because the temperature dependencies does not change the topological charge of the directors^41^ $\hat{n}=\hat{\rho }$ and $\hat{n}=\hat{\theta }$, keeping consequently^48^ the working modes of our proposed controller unchanged.

Controlling the amount of light and heat in a given region presents good possibilities for technological applications, for example, the energy saving by reuse of the heat produced secondarily by main physical systems, for use in other systems of interest. Another advantage of the proposed multiphysics controller is the spatial miniaturization on the development of energy manipulation projects, since a single artifact will act as a driver for different types of energy (light and heat).
