# Publisher Correction: Concurrent guiding of light and heat by transformation optics and transformation thermodynamics via soft matter

**DOI:** 10.1038/s41598-018-32599-5

**Published:** 2018-09-21

**Authors:** Wallysson K. P. Barros, Erms Pereira

**Affiliations:** 1 Polytechnic School of Pernambuco, Universidade de Pernambuco, Rua Benica, 455, 50720-001 Recife, PE Brazil; 2 Departament of Physics, Universidade Federal Rural de Pernambuco, 52171-900 Recife, PE Brazil

Correction to: *Scientific Reports* 10.1038/s41598-018-29866-w, published online 30 July 2018

An error occurred during the publication of this Article resulting in the inadvertent omission of the Article and Volume numbers and the publication date from the PDF version of this Article. In addition, maths symbols in the Abstract of the PDF version of this Article were incorrectly rendered. These errors have now been corrected in the PDF version of the Article; the HTML version was correct from the time of publication.

